# Population Dynamics and Body Size Structure of the Antarctic Krill *Euphausia superba* in the Bransfield Strait and South Shetland Islands

**DOI:** 10.3390/biology14111561

**Published:** 2025-11-07

**Authors:** Guoqing Zhao, Shuai Li, Jialiang Yang, Gangchen Zhang, Bo Xu, Hewei Liu, Xin Rao, Peng Lian, Hongliang Huang, Lingzhi Li

**Affiliations:** 1 East China Sea Fisheries Research Institute, Chinese Academy of Fishery Sciences, Shanghai 200090, China; zgq617717@163.com (G.Z.); lishuaiv@126.com (S.L.); yangjl@eastfishery.ac.cn (J.Y.); hwliu77@126.com (H.L.); raoxin128@gmail.com (X.R.); ecshhl@163.com (H.H.); 2 Key Laboratory of Polar Ecosystem and Climate Change (Shanghai Jiao Tong University), Ministry of Education, Shanghai 200030, China; 3 State Key Laboratory of Estuarine and Coastal Research, East China Normal University, Shanghai 200241, China; gczhang@sklec.ecnu.edu.cn; 4 Antarctic Great Wall Ecology National Observation and Research Station, Polar Research Institute of China, Shanghai 200136, China; xubo@pric.org.cn; 5 Chinese Academy of Fishery Sciences, Beijing 100141, China; lianpeng@cafs.ac.cn

**Keywords:** Antarctic krill, age, density, GAM, CCAMLR, climate change, krill fishery management

## Abstract

**Simple Summary:**

Antarctic krill (*Euphausia superba*) is a key species in the marine ecosystem of the Antarctic Ocean, with important ecological and economic value. This study used 6 years of midwater trawl data (including over 160,000 krill length measurements) to study the spatio-temporal changes and population composition of Antarctic krill, aiming to provide useful advice for better fishery management. We found that the krill fishing grounds are moving southward, and smaller krill prefer ice-rich areas in southern latitudes. Commercial fishing targets high-density krill areas rather than choosing larger krill. Importantly, the increase in fishing efforts in recent years has not made krill smaller. Environmental factors, geographical location, and the density of Antarctic krill swarms all exhibit nonlinear relationships with the average body length of Antarctic krill, and these factors all significantly affect its average body length. Our study is of great significance for understanding the population dynamics of Antarctic krill in the waters of the Antarctic Peninsula.

**Abstract:**

Antarctic krill (*Euphausia superba*) is a keystone species in the marine ecosystem of the Antarctic Ocean, bringing about significant ecological and economic value. The spatio-temporal distribution of Antarctic krill directly affects commercial fishing; meanwhile, changes in krill population structure play a crucial role in maintaining the balance of the Southern Ocean ecosystem. This study analyzed six years of midwater trawl data, including over 160,000 krill length measurements, to elucidate spatio-temporal dynamics and population composition, providing actionable insights for improved fishery management. Here, we reveal southward migration shifts in krill fishing grounds, with smaller individuals favoring ice-rich southern latitudes. Commercial krill fishing operations preferentially targeted high-density fishing grounds rather than selecting larger individuals. Among the catches, the age 1+ class accounted for the highest proportion at 42.80%, followed by the age 2+ class at 39.42%, with individuals ≥3+ accounting for 17.44%. Although the mean krill length experienced a decline in 2017, it demonstrated a sustained recovery in subsequent years, reaching peak dimensions in 2022. This maximum-growth year also exhibited the highest proportion (12.6%) of individuals within ≥4 age classes. Consequently, the sustained increase in fishing effort in recent years has not resulted in a reduction in the size of individual krill. The mean krill length showed a significant positive correlation with the depth (r = 0.36, *p* < 0.01) and temperature (r = 0.26, *p* < 0.01) of the krill cluster, and a significant negative correlation with resource density (r = −0.20, *p* < 0.01), year (ρ = −0.31, *p* < 0.01) and latitude (ρ = −0.31, *p* < 0.01). The length exhibited U-shaped temporal trends, and latitudinal and longitudinal nonlinearity. Body size was positively correlated with depth (*p* < 0.01), whereas as temperature increased, body size first increased and then remained constant. As density increased, the mean krill length increased first and then slowly decreased. Recent warming intensifies population shifts, with potential cascading effects on ecosystem structure and carbon sequestration.

## 1. Introduction

Antarctic krill (*Euphausia superba*, hereafter krill), a keystone species in the Southern Ocean, plays an important role in the food-web [1,2], mainly because it is a major component of the diet of marine mammals, birds, and fishes [3,4], and it also contributes to biogeochemical cycles [5,6] and carbon flux in the Southern Ocean [7,8,9]. Krill is one of the largest and longest-lived epipelagic zooplankton in the world, with an average length of more than 60 mm and a lifespan of more than six years [9,10]. They are widely distributed in the waters around Antarctica, but mostly abundant in the Atlantic sector of the Southern Ocean, where the biomass of krill resources account for approximately 30% of the total [11,12].

**Figure 1:**
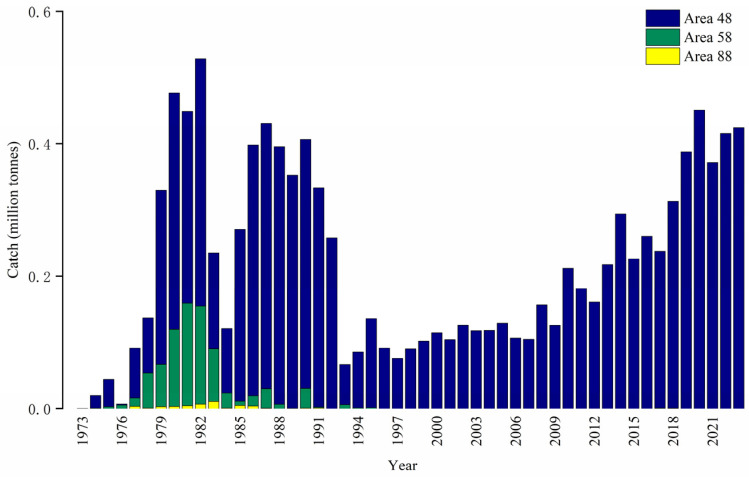
Total catch of Antarctic krill in area 48, 58, and 88 from 1973 to 2023. Note: Areas 48, 58, and 88 refer to management areas designated by CCAMLR, which are used for the management and statistics of Antarctic fishery resources.

Krill are one of the most abundant metazoan species in the world [13], whose biomass has been estimated at between 300 and 500 million tonnes in Antarctic waters [11], and it is also the main target species in the largest fishery in the Southern Ocean [14]. The krill commercial fishery started in the early 1970s [15], which has been going on for more than 50 years, with great fluctuation in the spatio-temporal distribution of fishing grounds and catches (Figure 1). Since the late 1990s, krill fishing activities have been almost entirely concentrated in the Antarctic Peninsula and South Shetland Islands (SSI), as well as South Georgia and the South Orkney Islands [16,17,18]. The Bransfield Strait (BS), located between the SSI and the West Antarctic Peninsula (WAP), is both an important spawning ground [19] and a major fishing ground [20,21].

The WAP is one of the areas under faster warming rates worldwide [22,23], with an average atmospheric temperature increase of 6 °C since 1950 [24], resulting in a continuous deterioration of sea ice coverage [25,26,27]. Climate warming and the reduction in the amount of sea ice may lead to a dramatic decrease in habitat quality [28,29,30] and the relative gross growth potential (RGGP) [31] of krill, thereby causing krill habitats and fishing grounds to shift to higher latitudes in the WAP [18,21,31]. Against the background of climate warming, most of the fishery management policies and studies focus on the changes in the biomass, abundance and distribution of krill resources [32,33,34], ignoring the composition and changes in the structure of krill populations, especially the relationship between the population structure and krill density. However, the size and population structure of krill should be given sufficient attention by managers.

Body length is very important for fishery management and resource assessment, because they can not only show the growth status of fish, but also be the key indicator to assess the status of fish population in some resource assessment models. However, it is worth noting that the size of krill has not been regarded as a management index in krill fishery management, which leads to our limited understanding of the growth of the krill population, especially its impact on the ecosystem. Studies have shown that krill is the main food source of penguins, which account for 100% of the diet of *Pygoscelis* penguins in the WAP [35]. Among them, gentoo and chinstrap penguins prefer larger krill, even though small krill individuals are dominant in their habitat waters [36]. Furthermore, the krill body size exerts a dominant control on the particulate organic carbon (POC) flux, which oscillates synchronously with krill body length [9]. Therefore, it is necessary to pay attention to the changes in the length of krill for the protection of ecology and the guidance of fishery production. Due to the lack of attention to krill body size in the past, it is a challenge for Commission for the Conservation of Antarctic Marine Living Resources (CCAMLR) to balance the relationships among the dynamics of krill biomass, fishing ground, and the population structure under the premise of climate warming.

The CCAMLR is the sole multilateral body responsible for managing marine living resources under the framework of The Antarctic Treaty. It undertakes key tasks such as formulating fishery resource conservation measures, reviewing fishing applications, and releasing statistical data. The Ecosystem-Based Quota Management System is currently the primary management approach for the Antarctic marine ecosystem [37,38,39]; for krill, this means setting differentiated quotas based on variations in krill biomass, natural resource fluctuations, and predator distribution (such as seabirds, seals, whales, and penguins) across different statistical subareas [40]. The sustainable development of the krill fishery is safeguarded through the implementation of fishery restrictions. These restrictions ensure that a sufficient quantity of krill remains after fishing operations, thereby maintaining an adequate spawning stock and providing sufficient prey for predators [41]. The sustainability of the krill fishery is contingent upon the size of the catch relative to the krill population [39]. However, the current krill management overlooks the impacts of changes in krill population structure on the entire ecosystem, which constitutes a highly research-worthy issue.

Based on midwater trawling data of 6 years, including more than 160,000 krill length data, this study analyzed the distribution of catches, density, length, and age of krill in the BS and SSI. Furthermore, we conducted additional analyses to elucidate the relationship between krill length and environmental variables using a Generalized Additive Model (GAM), specifically examining how morphological variations in krill populations correlate with gradients in key abiotic factors across sampled habitats. This study found that there was a correlation between the krill body size and the depth and temperature of the water layer of krill cluster, krill density, year, month, latitude, and longitude. Although there is no direct evidence at present that the size of krill has a negative impact on the ecosystem, whether it should be added into fishery management needs to be considered carefully by CCAMLR.

## 2. Materials and Methods

### 2.1. Data Resources

**Figure 2:**
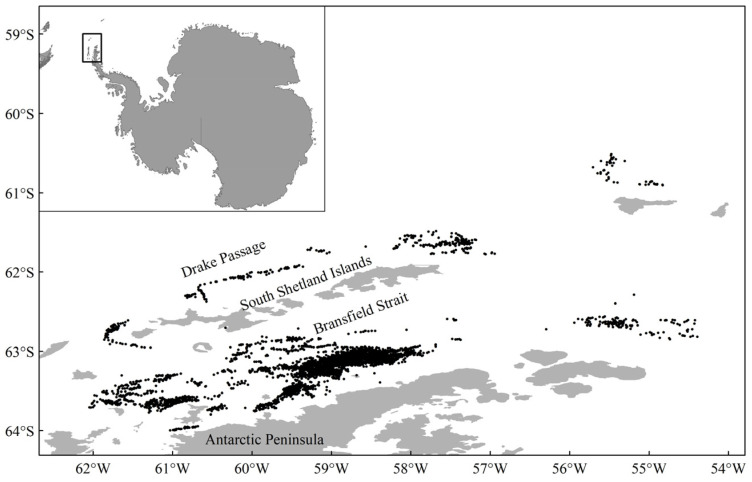
Locations of commercial fishing stations of the Antarctic krill in the CCAMLR 48.1 subarea.

The commercial fishery data and the length data of krill came from BS and SSI in the CCAMLR 48.1 subarea, the major krill fishing grounds (Figure 2). The krill fishery data in this paper included a total of 4491 hauls from 2016 to 2022, including the time of starting and ending the trawling, catches, longitude, latitude, depth and temperature of the water layer of krill cluster. However, it should be noted that there was a data gap in 2020 due to the COVID-19 outbreak. A total of 811 hauls were randomly selected, and 200 krill were randomly selected from each haul to measure their body length, with a total of 162,200 krill individuals measured in total length (Table 1). The total length of krill was measured from the anterior margin of the eyeball to the tip of the telson [42], following the CCAMLR standard protocol (https://www.ccamlr.org/, accessed on 5 May 2025). The krill length data were accurate to 0.1 mm. The depth (DepKRI) and the temperature (TempKRI) of krill cluster centers were measured using a temperature–depth recorder attached to the trawl.

### 2.2. Statistical Analysis

In order to visualize the spatial distribution of krill resources and krill body size more clearly, the data was standardized to 0.1° × 0.1°. The Kolmogorov–Smirnov test shows that length, resource density, CPUE, catch, and the temperature and depth of the krill cluster’s water layer all conform to a normal distribution. Additionally, year, month, longitude, and latitude obviously do not conform to a normal distribution. Therefore, Pearson correlation analysis was performed on krill length, resource density, CPUE, catch, the temperature and depth of the water layer of krill cluster, while Spearman correlation analysis was used to examine correlations between the krill length, year, month, longitude, and latitude. When performing correlation analysis, the krill length was the average length of each haul. The age of krill was divided according to the standards of Candy and Kawaguchi [5]: Age 0: 0–25.9 mm, Age 1+: 26–39.9 mm, Age 2+: 40–47.9 mm, Age 3+: 48–50.9 mm, Age 4+: 51–52.9 mm, Age 5+: 53–54.9 mm, and Age 6+: 55–70 mm.

In commercial fisheries’ stock assessments, CPUE has been widely adopted as a pivotal proxy for relative abundance, serving to elucidate fishery population dynamics and quantify the ecological impacts of fishing pressure on regional stock structures [43]. However, despite ongoing debates regarding the validity of CPUE as a reliable abundance indicator in Antarctic krill fisheries [44], its spatio-temporal datasets retain utility in characterizing distribution heterogeneity and identifying temporal persistence patterns within localized krill populations [17,45]. To improve the accuracy of krill resource abundance estimation, this study used both CPUE and density as indicators of krill abundance and conducted comparative analyses to identify the indicator that more accurately reflects krill resource abundance. The computational formulations of these two indices are defined as follows:

CPUE = C/T(1)

Density = C/(V × 60 × T × W)(2)

where C is the catch (t) of per haul; T (h) is the trawling time of per haul; V (m/s) is the speed of the trawling; W (m) is the horizontal expansion distance of the net mouth.

### 2.3. Gravity Center of Fishing Ground

The gravity center analysis method quantifies spatio-temporal dynamics of fishing grounds across multiple temporal resolutions by statistically aggregating catch-weighted geographic coordinates. This geospatial approach effectively reduces high-dimensional fishing effort data into interpretable trajectory vectors, while preserving essential spatial heterogeneity information. This spatially explicit metric, calculated through the following equation, enables systematic tracking of fishery resource distribution patterns [46]:

(3) $$X_{w}=\frac{\sum_{i=1}^{n}(C_{i}\times X_{i})}{\sum_{i=1}^{n}C_{i}}$$

(4) $$Y_{w}=\frac{\sum_{i=1}^{n}(C_{i}\times Y_{i})}{\sum_{i=1}^{n}C_{i}}$$

where *X_i_* and *Y_i_* represent the latitude and longitude of each fishing net, *X_w_* and *Y_w_* represents the coordinates of the center of the fishing ground gravity; *C_i_* represents the yield of net *i*; *n* represents the total number of nets in the statistical period.

### 2.4. Generalized Additive Model (GAM)

In this study, GAM was used to analyze the spatial distribution of krill length in BS and SSI. Based on the results of the correlation analyses in Section 3, the average length of krill was considered as the response variable, while the year, month, longitude, latitude, density, DepthKRI and TempKRI were considered as the spatial and temporal explanatory variables. GAM was constructed as follows:

log(Length)~s(Year) + s(Month) + s(Longitude) + s(Latitude) + s(DepthKRI) + s(TempKRI) + s(Density) + ε(5)

where length is the response variable; s(year) is the spatio-temporal explanatory variable year; s(month) is the spatio-temporal explanatory variable month; s(longitude) is the spatio-temporal explanatory variable longitude; s(latitude) is the spatio-temporal explanatory variable latitude; s(density) is the explanatory variable density of krill; s(DepthKRI) is the environmental explanatory variable depth of krill cluster; s(TempKRI) is the environmental explanatory variable temperature of krill cluster; and ε is the random error term.

The variance inflation factor (VIF) was used to test the independence of the explanatory variables, and the results are shown in Table 2. Predictor variables demonstrated acceptable collinearity (VIF < 4), consistent with established diagnostic thresholds for regression models [47,48]. This conservative criterion minimizes Type I errors while maintaining model parsimony, as justified by Monte Carlo simulations in ecological studies [49]. Based on the results of VIF test (Table 2), the VIF month value was more than 4, indicating that there was no serious multicollinearity between the explanatory variables.

The model utilized a stepwise regression framework to select predictors by optimizing the Akaike Information Criterion (AIC) [50] and the bias explanation rate [51]. Improved model fit was indicated by lower AIC values and higher bias explanation rate [48,52]. The model was implemented using the package mgcv in R 4.4.3.

## 3. Results

### 3.1. The Spatio-Temporal of Green Weight, CPUE and Density of Commercial Catches of the Antarctic Krill

**Figure 3:**
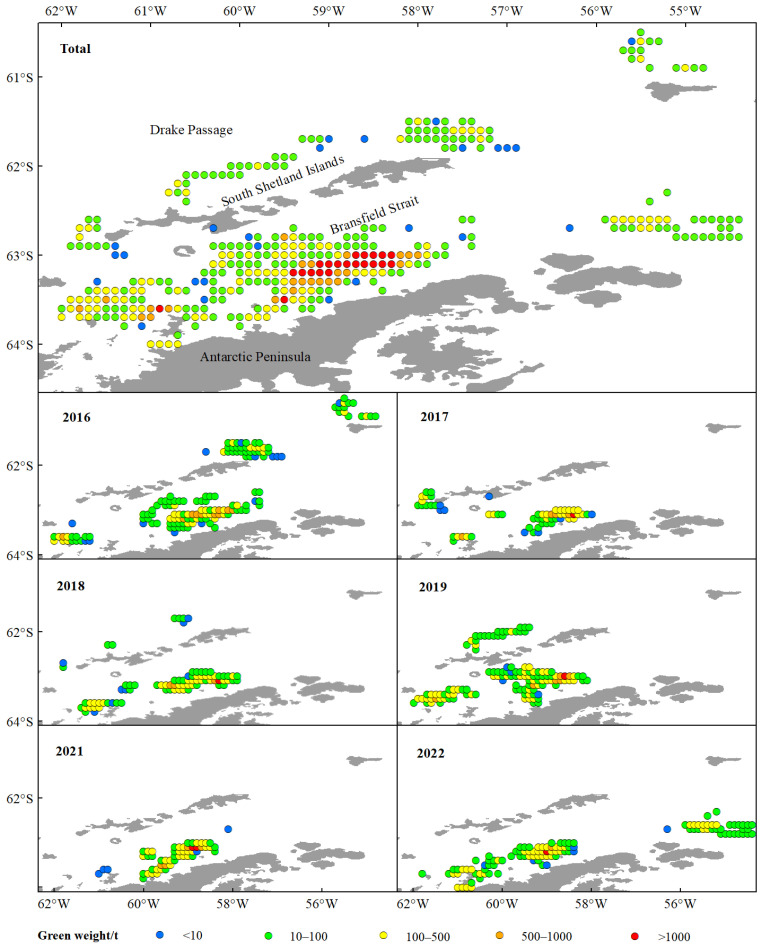
The spatio-temporal dynamics of green weight of commercial catches of the Antarctic krill in BS and SSI from 2016 to 2022. Note: The color codes indicate green weight ranges as follows: blue for <10 t; green for 10~100 t; yellow for 100~500 t; orange for 500~1000 t; red for >1000 t.

**Figure 4:**
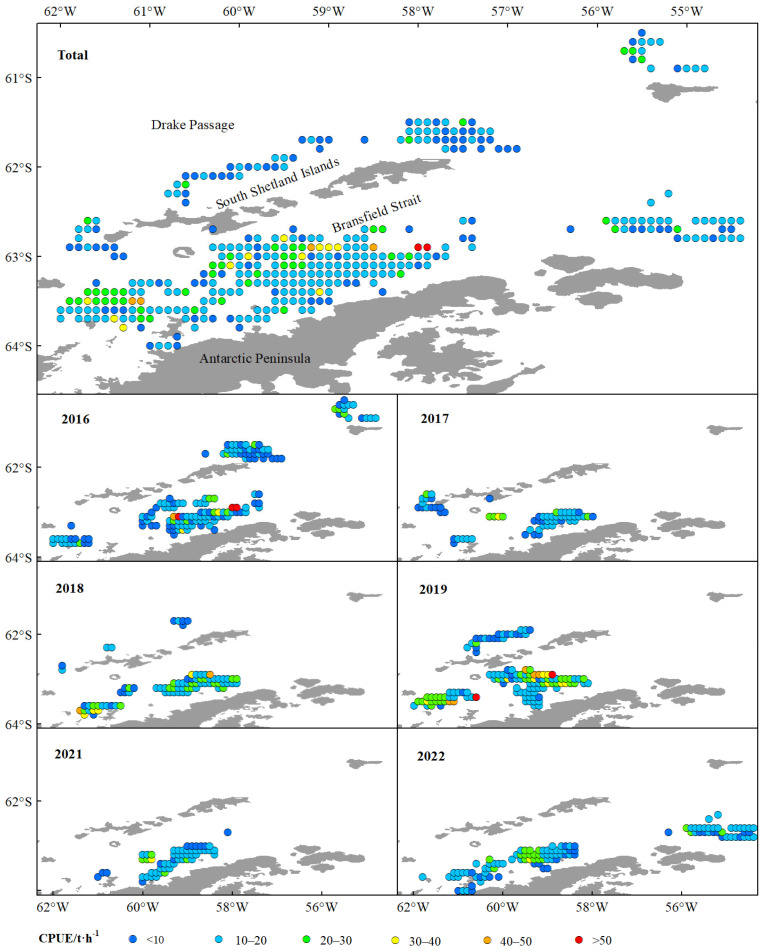
The spatio-temporal dynamics of CPUE of commercial catches of the Antarctic krill in BS and SSI from 2016 to 2022. Note: The color codes indicate CPUE ranges as follows: blue for <10 t·h^−1^; baby blue for 10~20 t·h^−1^; green for 20~30 t·h^−1^; yellow for 30~40 t·h^−1^; orange for 40~50 t·h^−1^; red for >50 t·h^−1^.

**Figure 5:**
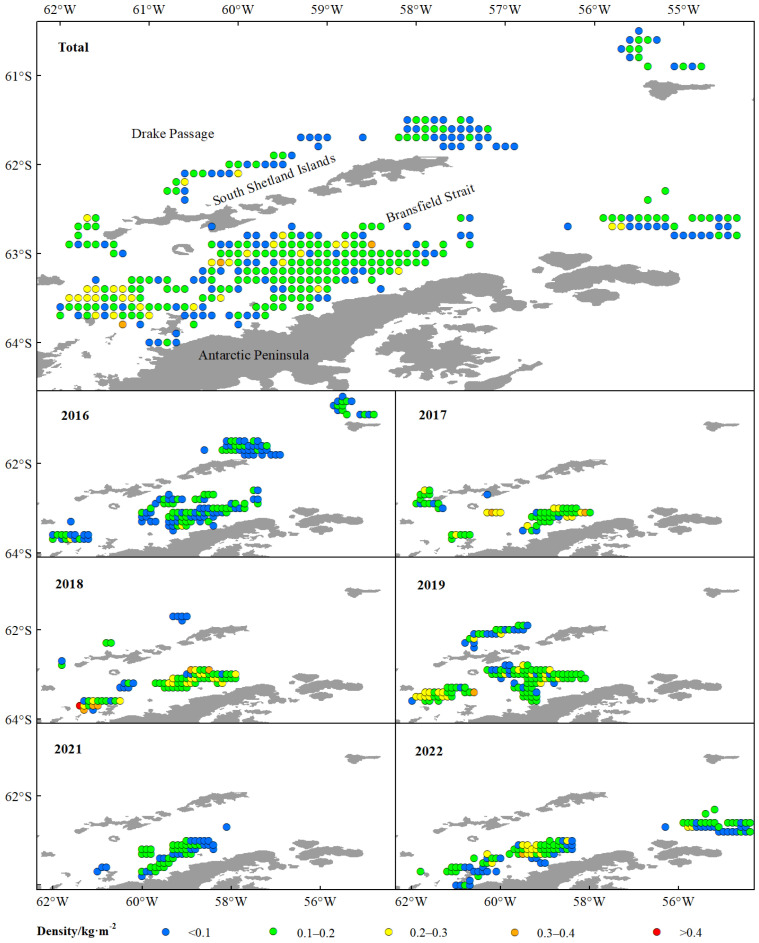
The spatio-temporal dynamics of density of the Antarctic krill in BS and SSI from 2016 to 2022. Note: The color codes indicate density ranges as follows: blue for <0.1 kg·m^−2^; green for 0.1~0.2 kg·m^−2^; yellow for 0.2~0.3 kg·m^−2^; orange for 0.3~0.4 kg·m^−2^; red for >0.4 kg·m^−2^.

**Figure 6:**
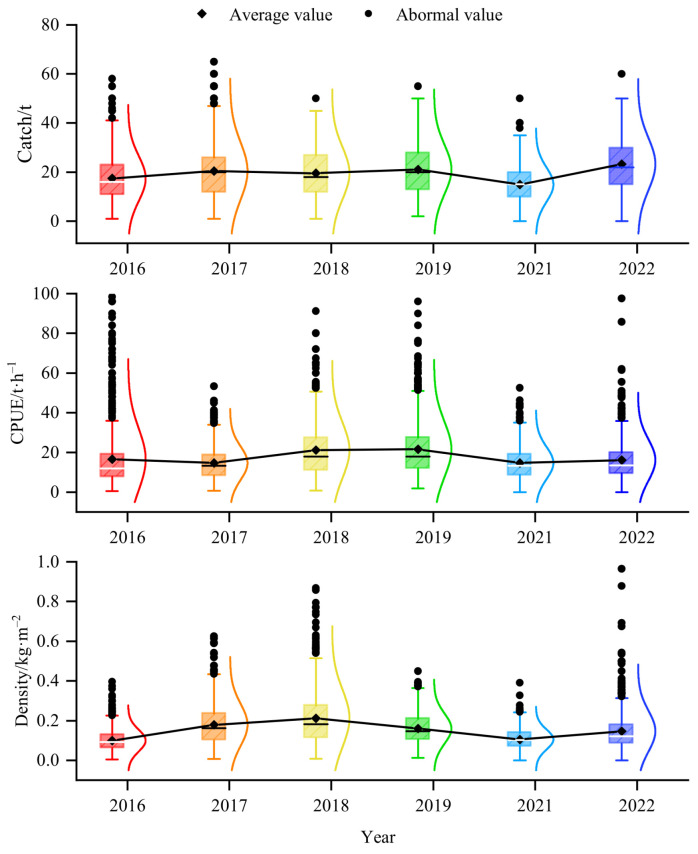
The distribution of the average value of catch, CPUE and density of the Antarctic krill from 2016 to 2022. Note: The upper edge and lower edge of the box in the figure indicate that 75% of the data are less than the value of the upper edge, and 25% of the data are less than the value of the lower edge, respectively. The height of the box is the difference between the third quartile (Q3) and the first quartile (Q1). The horizontal line in the middle of the box represents the median. The whiskers are the lines extending from the upper and lower edges of the box, representing the normal fluctuation range of the data, and this figure uses the Tukey method to define the range. The polyline in the figure is the average value line. The curve on the right side of the box is the normal curve for each group of data.

**Figure 7:**
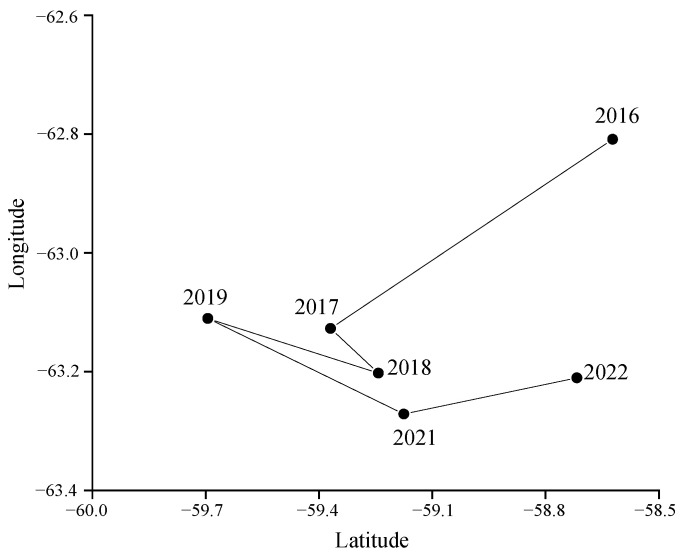
The center gravity of the krill fishing ground.

The green weight of krill throughout the study was greater in the BS than in the Drake Passage, as well as the fishing effort (Figure 2), and the highest green weight was concentrated in the southern part of the BS, near the continental shelf of Antarctic Peninsula (Figure 2 and Figure 3). Most of the fishing activities were close to the continental shelf, and the fishing grounds had a trend of moving southward year by year. In general, the most of high values of CPUE and density distributed in the BS, rather than in the Drake Passage (Figure 4 and Figure 5). The spatio-temporal distribution of the high values of density, which were similar to the catch, were relatively more concentrated compared with CPUE value. From 2016 to 2022, the average values of catch, CPUE, and density fluctuated, but the changes were not significant (Figure 6). The krill fishing grounds exhibited a southward-shifting trend (Figure 7).

### 3.2. The Spatio-Temporal Distribution of Body Size and Age of the Antarctic Krill

**Figure 8:**
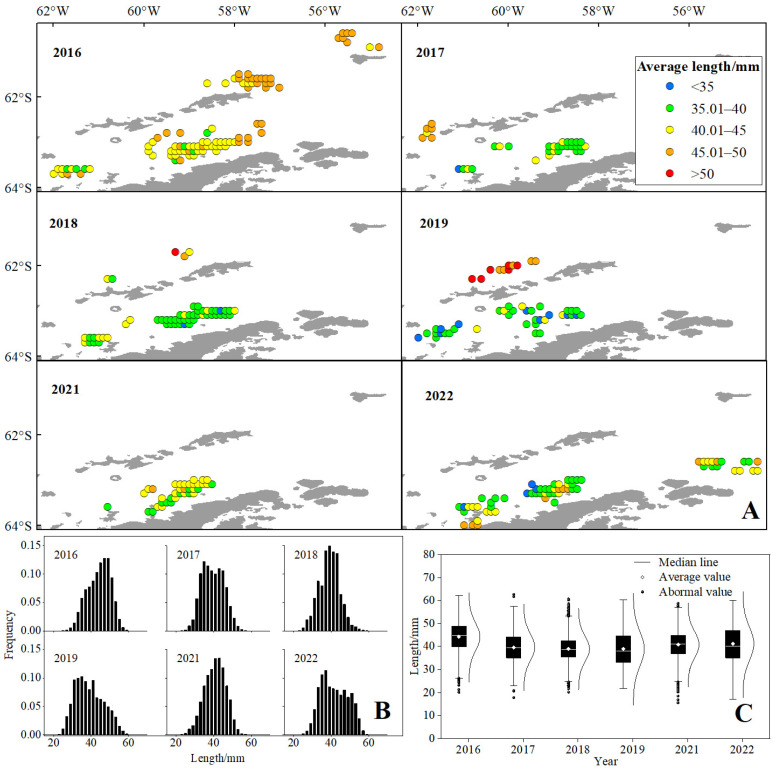
The spatio-temporal dynamics of average length (**A**), length frequency distribution (**B**) and distribution interval of length (**C**) of the Antarctic krill from 2016 to 2022. Note: The color codes indicate average length ranges as follows: blue for <35 mm; green for 35.01~40 mm; yellow for 40.01~45 mm; orange for 45.01~50 mm; red for >50 mm.

The spatio-temporal distribution of krill average length is shown in Figure 8 and Table 3. Spatially, the largest krill (>45 mm) were observed at the continental shelf boundary north of the SSI, where larger individuals dominated. The smaller krill were concentrated throughout the entire BS, where the krill population was diverse, but small krill (<40 mm) dominated (Figure 8A). The length frequency distribution of krill showed that there was one peak in all years (Figure 8B), and the average length decreased first and then increased by years (Figure 8C). There was a significant difference in krill length among different years (Table 4), and relatively large krill (>45 mm) dominated only in 2016.

**Figure 9:**
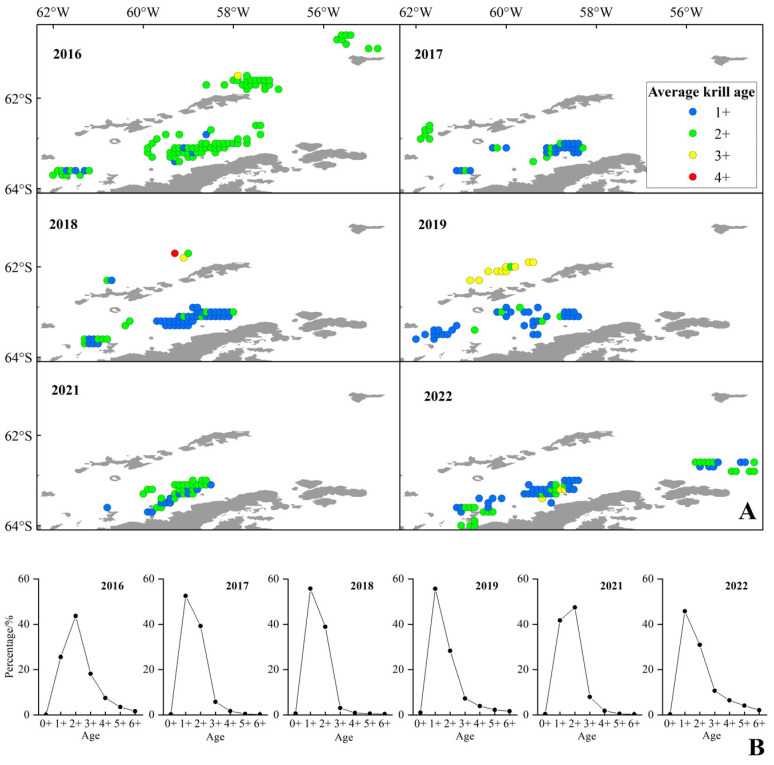
The distribution of age (**A**) and age frequency (**B**) of the Antarctic krill from 2016 to 2022. Note: The color codes indicate average krill age ranges as follows: blue for 1+; green for 2+; yellow for 3+; red for 4+.

The spatial distribution of the age of krill was significantly different, 2+ and 3+ krill dominated north of the SSI, whereas the 1+ and 2+ krill dominated in the BS (Figure 9A). In terms of years, 2+ krill dominated in 2016 and 2021, while 1+ krill dominated in other years (Figure 9B), and 1+ krill dominated in the BS except for 2016 and 2021. Among all years surveyed, the proportion of krill aged 4+ and above was highest in 2022, accounting for 12.6% of the population. Consequently, substantial quantities of juvenile Antarctic krill (1+ and 2+ year classes) were captured within the CCAMLR Area 48.1, accounting for 82.2% of the total samples (Figure 9B).

### 3.3. Correlation Analysis

The results of correlation test are shown in Table 5. The average length of krill had a significant positive correlation with the depth (r = 0.36, *p* < 0.01) and temperature (r = 0.26, *p* < 0.01) of krill cluster, and a significant negative correlation with krill density (r = −0.22, *p* < 0.01), (ρ = −0.31, *p* < 0.01) and latitude (ρ = −0.31, *p* < 0.01), a weak negative correlation with month (ρ = −0.12, *p* < 0.01). The krill density had a significant positive correlation with CPUE (r = 0.50, *p* < 0.01), catch (r = 0.76, *p* < 0.01) and year (ρ = 0.31, *p* < 0.01), and a weak negative correlation with the depth (r = −0.15, *p* < 0.01) and temperature (r = −0.13, *p* < 0.01) of krill cluster.

### 3.4. GAM Analysis

#### 3.4.1. GAM Test

**Figure 10:**
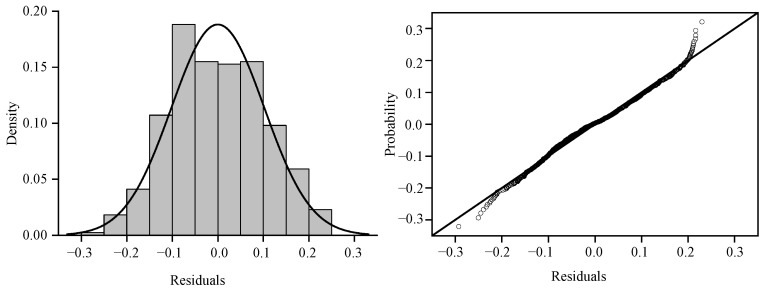
The diagnostic analysis of residuals.

As summarized in Table 6, the optimal GAM demonstrated robust performance, with an AIC of 2018.369, 54.6% deviance explained, and an R^2^ value of 0.535. To validate the model’s generalizability, residual diagnostics and spatial blockwise cross-validation were rigorously applied. Residual analysis revealed no systematic patterns or heteroscedasticity, alongside a stable effective degree of freedom (edf) trajectory and declining AIC, consistent with appropriate regularization (Figure 10). These results collectively demonstrate that GAM, optimized through iterative refinement, avoids overfitting and reliably captures underlying data structures.

The statistical significance of variables in the final optimized GAM is presented in Table 7. Results from the F-test analysis revealed that all predictors, including interaction terms, demonstrated statistically significant associations with length measurements (*p* < 0.05).

#### 3.4.2. Distribution of Length Under Different Factors

**Figure 11:**
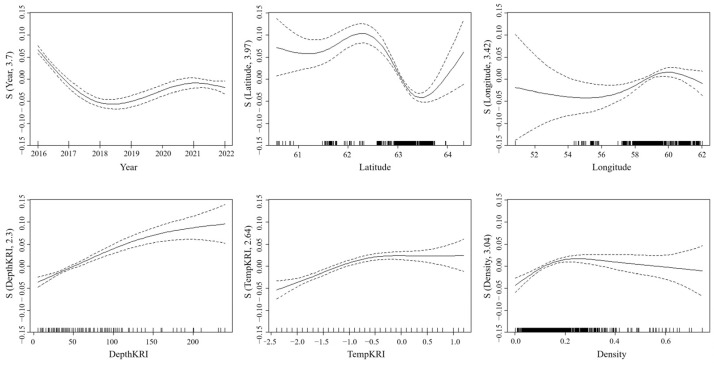
The impact of each explanatory variable on the average length of Antarctic krill in BS.

The effects of different predictors on Antarctic krill length are illustrated in Figure 11. During the period 2016–2022, the average length showed a downward trend, followed by a rebound, with the minimum occurring in 2018. Latitude displayed a nonlinear relationship with length, characterized by an initial increase (61~62.5° S), followed by a decline (62.5~63.5° S), and then increase (63.5~64.5° S). The average length of Antarctic krill exhibited a three-phase longitudinal pattern: stabilization between 51° W and 56° W, followed by an increase from 56° W to 60° W, and ultimately a decrease between 60° W and 62° W. Notably, depth exhibited a significant positive linear relationship with length (*p* < 0.001), implying enhanced growth potential in deeper water layers (>200 m), likely associated with reduced predation pressure. Temperature dependence followed a threshold response: length increased with temperature within the range of −2.5 °C to 0 °C before plateauing above 0 °C, consistent with metabolic optimization under moderate thermal conditions. As the density increases, the mean krill length initially increases (0~0.2 kg/m^2^) and then decreases (>0.2 kg/m^2^), with the rate of increase being greater than the rate of decrease.

## 4. Discussion

The BS and the SSI constitute the core distribution area of Antarctic krill and a hotspot for commercial fishing [20]. However, dramatic environmental changes in the region in recent years have raised significant ecological concerns: sea surface temperature (SST) has continued to rise at a rate of 0.31 °C per decade since the beginning of the 21st century [53], and the extent of winter sea ice cover has shown a significant contraction since 2016 [54]. In the Southwest Atlantic sector, Antarctic krill distribution has exhibited a persistent southward contraction over the past nine decades. Along its northern boundary, population density has undergone a sharp decline, with the stock becoming increasingly concentrated on the Antarctic continental shelf [18,21]. Under this climatic regime, commercial fishing grounds have demonstrated progressive poleward latitudinal shifts [18,21,55], mirroring krill population redistribution toward higher latitudes where remnant sea ice persists. The southward shift of the Antarctic krill fishing ground’s center of gravity is the most intuitive response to climate warming, which has led to the southward movement of krill’s suitable habitats [55] and will also exert a significant impact on the Antarctic marine ecosystem. The southward shift of the krill fishing ground’s center of gravity is bound to increase fishing effort, which in turn will raise the pressure on the marine ecosystem in this sea area to maintain biodiversity. Therefore, incorporating climate-driven changes in krill habitats into the sustainable fishery management framework is crucial.

This study showed that krill green weight and fishing effort were significantly higher in the BS than in the Drake Passage, and that the area of high green weight values was concentrated in the southern end of the BS adjacent to the continental shelf of the Antarctic Peninsula (Figure 2 and Figure 5). This spatial pattern is closely linked to the habitat preferences of Antarctic krill, as continental shelf margins and upwelling zones typically exhibit higher primary productivity, providing abundant food resources for krill [56,57]. Further analysis indicated that the high-value areas of CPUE and krill density were primarily distributed in the BS region rather than the Drake Passage (Figure 4 and Figure 5). This difference may originate from the more stable hydrological conditions (e.g., circulation systems) and higher resource aggregation in the BS, which significantly enhance fishing efficiency [14]. Quantitatively, the correlation between krill swarm distribution and catch volume (r = 0.767) was significantly stronger than that between CPUE and catch (r = 0.505) (Figure 6, Table 5), validating density as more reliable indicators of fishable aggregations than effort-adjusted catch rates. This inconsistency may reflect the sensitivity of CPUE data to fishing strategies (e.g., dynamic adjustments of fishing vessel operations), whereas density estimates more directly reflect the actual distribution of biomass [58,59].

Although there were no statistically significant differences in mean annual fluctuations in catch, CPUE, and density between 2016 and 2022 (Figure 6), trends in the southward shift of the fishery (Figure 9) suggest potential climate-driven mechanisms. For example, rapid warming (0.31 °C/decade) [53] in the waters off the western side of the Antarctic Peninsula, coupled with plummeting winter sea ice cover (~20% reduction after 2016) [54], may indirectly affect krill distribution boundaries by altering plankton community structure or ice algal productivity. It is crucial to note that food competition and SST anomalies may drive reductions in Antarctic krill abundance, coupled with their southward shifts toward the Antarctic Peninsula or migration to deeper, colder habitats [60]. Furthermore, environmental perturbations—such as SST anomalies or shifts in water masses across depth gradients—could act as indirect drivers of ecosystem destabilization, altering the composition and biomass of zooplankton communities in regions like the Bransfield Strait [61]. However, the time span of the current data (6 years) may not be sufficient to capture long-term trends, and longer time series of environmental and harvest data need to be combined to verify causal associations.

In this study, the spatial and temporal heterogeneity of krill body length distributions and its driving mechanisms were systematically resolved for the first time by a GAM based on 6 years of mesocosm trawl data (covering 160,000 individual body length measurements) from BS and SSI. The results of model residual analysis and spatial cross-validation showed that the GAM was able to effectively capture nonlinear environmental responses and provided a reliable tool for resolving complex ecological relationships. The results showed that the spatial distribution of krill body length showed significant nonlinear characteristics, with an extreme point at 62.5° S on the latitudinal gradient (*p* < 0.01), while the longitudinal extremes were concentrated around 60° W. This distribution pattern may be closely linked to regional ocean dynamical processes: the 57° W sea area is located at the entrance to BS on the eastern side of the SSI, which is subject to the interaction of the Antarctic Circumpolar Current branch with land-slope upwelling to form a highly productive frontal system [62,63,64], which provides a steady supply of bait for krill clusters. At the same time, the peak body length on the latitudinal gradient (62° S) was highly coincident with the edge of summer sea ice retreat, supporting an adaptive strategy of small individuals to the ecological niche of the ice-marginal zone. It is noteworthy that the mean body length of krill individuals caught in the high-latitude region (>63° S) was lower than in the northern fishing ground, which may be related to the dependence of larvae on sea ice microhabitats (e.g., ice algal resources) [65]. Positive correlations of krill body length with depth (r = 0.36, *p* < 0.01) and temperature (r = 0.26, *p* < 0.01) reveal the ecological advantages of the deep-water layer for large individuals, where lower predation pressure and a stable thermal environment in deeper water may promote individual survival and growth [66]. Aggregation of large individuals in deeper water may be related to a gradient in predation pressure, and reduced light availability in deeper waters reduces the predation efficiency of seabirds and cetaceans. The negative correlation between krill body length and density (r = −0.22, *p* < 0.01), which was similar to the results of [9], and its “parabola-shaped” density-dependent curve, reflected the dynamic balance between intra-population competition and resource availability. In low-density areas (<0.2 kg/m^2^), the abundance of resources may promote individual growth. However, when population density exceeds this threshold, adult individuals likely achieve ecological niche partitioning through vertical migration to deeper water layers.

While this study elucidates multiscale drivers of Antarctic krill size distribution, the following limitations warrant consideration: 1. Temporal Resolution Constraints. The six-year dataset remains insufficient to resolve long-term climate oscillation effects. Decadal-scale mechanistic analysis requires the integration of historical specimen archives spanning the climate phases of more years. 2. Vertical Migration Mechanism Gaps. Trawl sampling biases toward krill-intensive areas and may have underestimated the biomass of large individual krill in deeper waters. Future efforts should incorporate underwater glider hydroacoustic surveys to quantify diel vertical migration-driven size stratification. 3. Multifactorial Interaction Oversights. The synergistic effects between sea ice coverage and climate change remain unquantified. Mechanistic disentanglement necessitates coupled biogeochemical models with ice-ocean atmosphere-forcing modules.

## 5. Conclusions

The key findings of this research are as follows: First, krill fishing grounds showed a significant southward-shifting trend, with smaller krill preferring ice-rich areas at higher southern latitudes, while commercial fishing targeted high-density krill areas rather than selecting larger individuals. Notably, the continuous increase in fishing effort in recent years did not lead to a reduction in krill body size. Second, the mean body length of krill had a significant positive correlation with the depth and temperature of krill clusters, and a significant negative correlation with density and latitude; body size also exhibited nonlinear relationships with latitude, longitude, and temperature (increasing first then stabilizing with temperature). Additionally, the krill population in the study area was dominated by juvenile individuals, with 1+ and 2+ age classes accounting for 82.2% of the total samples. The southward shift of Antarctic krill fishing grounds caused by climate warming should be given sufficient attention, as it exerts a significant impact on the Antarctic marine ecosystem. CCAMLR should incorporate this situation into its ecological management framework to achieve the goals of protecting the Antarctic marine ecology and promoting the sustainable development of the krill fishery.

This study provides critical insights into the population dynamics of Antarctic krill in the BS and SSI waters, and highlights that environmental factors, geographical location, and krill density jointly shape krill body size and population structure. Given the ecological importance of krill body size and the ongoing climate warming in the Antarctic Peninsula, it is recommended that the CCAMLR consider incorporating krill body size into fishery management indicators to balance the sustainability of krill fisheries and the stability of the Southern Ocean ecosystem. We must point out that changes in the krill population structure require longer-term data accumulation. Additionally, it is also necessary to incorporate other indicators, such as sea ice coverage rate and ocean currents, to conduct more in-depth research.

## Figures and Tables

**Table 1:** The information from samples.

Year	Month	Vessel	Number of Hauls	Number of Measured Krill
2016	2~5	Furonghai	1084	46,200
2017	3~6	Longteng	517	19,600
2018	2~5	Longteng	704	26,800
2019	3~7	Longteng	857	21,000
2021	3~6	Furonghai	763	25,600
2022	3~6	Longfa	566	23,000

**Table 2:** Variable VIF testing results.

Variable	Year	Month	Longitude	Latitude	DepKRI	TempKRI	Density
VIF value	1.965	4.414	2.422	2.433	1.379	3.628	2.724

**Table 3:** Basic statistics on the size of the Antarctic krill over six years in the BS.

Year	Number	Mean	SD	Med.	Mode	Min.	Max.	Q25	Q50	Q75	Skew	Kurt
2016	46,200	44.16	5.97	44.83	47.6	20.1	62.2	39.76	44.83	48.75	−0.288	−0.595
2017	19,600	39.62	5.80	39.38	35.41	17.77	62.69	35.01	39.38	44.16	0.109	−0.672
2018	26,800	38.91	5.46	38.7	43.6	20.19	60.67	35.4	38.7	42.52	0.025	0.061
2019	21,000	38.94	7.50	38	40	21.78	60.39	33	38	44.62	0.35	−0.718
2021	25,600	40.79	5.61	41.1	42.4	15.6	58.7	36.83	41.1	44.9	−0.184	−0.312
2022	23,000	41.14	7.18	40	37	17	60	35	40	47	0.16	−0.964

**Table 4:** Results of ANOVA and Tukey’s post hoc tests comparing the body sizes of the Antarctic krill over six years.

Year	diff	lwr	upr	*p* adj
2016–2017	4.54044	4.3902	4.6907	0.000
2016–2018	5.25227	5.1169	5.3876	0.000
2016–2019	5.21061	5.0525	5.3687	0.000
2016–2021	3.36452	3.2272	3.5019	0.000
2016–2022	3.01439	2.8722	3.1566	0.000
2017–2018	0.71183	0.5462	0.8775	0.000
2017–2019	0.67017	0.4854	0.8549	0.000
2017–2021	−1.17592	−1.3432	−1.0086	0.000
2017–2022	−1.52605	−1.6974	−1.3547	0.000
2018–2019	−0.4166	−0.5145	−0.2312	0.000
2018–2021	−1.88775	−2.0418	−1.7337	0.000
2018–2022	−2.23788	−2.3963	−2.0179	0.000
2019–2021	−1.34609	−1.4905	−1.1717	0.000
2019–2022	−1.79622	−1.9545	−1.6379	0.000
2021–2022	−0.35013	−0.5103	−0.1900	0.000

ANOVA *p* < 0.05.

**Table 5:** Basic statistics describing the differences in the size of the Antarctic krill between six years in BS.

Correlation	Length	Density	CPUE	Catch	DepKRI	TempKRI	Year	Month	Latitude	Longitude
Length	—	−0.221 **	−0.011	−0.020	0.360 **	0.261 **	−0.314 **	−0.125 **	−0.369 **	−0.179 **
Density	−0.221 **	—	0.505 **	0.767 **	−0.150 **	−0.134 **	0.311 **	0.093 **	0.0370	0.042
CPUE	−0.011	0.505 **	—	0.551 **	−0.104 **	−0.168 **	0.022	0.038	−0.050	−0.045
Catch	−0.020	0.767 **	0.551 **	—	−0.066	−0.199 **	0.110 **	0.101 **	−0.028	−0.022

Note: ** represent *p* < 0.01.

**Table 6:** GAM selection based on AIC.

GAM	R^2^	AIC	Explanation Rate (%)
log(Length)~s(Year)	0.285	−1695.017	28.8
log(Length)~s(Year) + s(Latitude)	0.411	−1843.158	41.6
log(Length)~s(Year) + s(Latitude) + s(Longitude)	0.445	−1887.595	45.3
log(Length)~s(Year) + s(Latitude) + s(Longitude) + s(DepKRI)	0.483	−1939.743	49.2
log(Length)~s(Year) + s(Latitude) + s(Longitude) + s(DepKRI) + s(TempKRI)	0.514	−1986.841	52.4
log(Length)~s(Year) + s(Latitude) + s(Longitude) + s(DepKRI) + s(TempKRI) + s(Density)	0.535	−2018.369	54.6

**Table 7:** ANOVA of the optimal GAM.

Parameter	df	F	*p* Value
Year	3.945	57.443	2 × 10^−16^
Latitude	3.998	24.298	2 × 10^−16^
Longitude	3.829	3.489	0.00777
DepKRI	2.803	31.062	2 × 10^−16^
TempKRI	3.168	19.450	2 × 10^−16^
Density	3.535	9.145	1.872 × 10^−6^

## Data Availability

The original contributions presented in the study are included in the article, and further inquiries can be directed to the corresponding authors.
